# Bacteria-Derived Recombinant Human ANGPTL8/Betatrophin Significantly Increases the Level of Triglyceride

**DOI:** 10.1007/s10930-019-09825-8

**Published:** 2019-03-30

**Authors:** Fangfang Xu, Yuqing Chen, Nan Wang, Kai Sun

**Affiliations:** 1 Department of Research and Discipline Development, Henan Provincial People’s Hospital, No. 7 Weiwu Road, Jinshui District, Zhengzhou, Henan 450003 China; 2 Biotechnology Research Institute, Chinese Academy of Agricultural Sciences, Beijing, 100081 China; 3 Department of Hematology, Henan Provincial People’s Hospital, Zhengzhou, 450003 China

**Keywords:** ANGPTL8/betatrophin, Recombinant expression, Beta cell proliferation, Triglyceride level

## Abstract

ANGPTL8/Betatrophin has been implicated in the regulation of both glucose and triglyceride metabolism. However, its role in regulating glucose metabolism by promoting β cell proliferation remains controversial, and its physiological functions and molecular targets are largely unknown. Hence, it is of great importance to make recombinant protein and test its effects on β cell mass directly. In this study, the mature form gene of human ANGPTL8/betatrophin was obtained through chemical synthesis on to the vector pUCE, and the fusion protein was expressed in the Transetta (DE3)/pEASY-E2-betatrophin strain. The inclusion bodies were solubilized in urea and purified by Ni–NTA affinity chromatography. The yield of purified ANGPTL8/betatrophin was approximately 20 mg per liter of culture medium. In vitro studies revealed that the recombinant ANGPTL8/betatrophin had no proliferation effect on MIN6 cells but promoted TG levels in HepG2 cells. This method to generate bioactive ANGPTL8/betatrophin is a simple, practical and user-friendly protocol.

## Introduction

Type 2 diabetes mellitus (T2D), characterized by hyperglycemia, results from dysfunctional carbohydrate metabolism that is caused by a relative deficiency of insulin. T2D has become a major health burden, with current prevalence estimated at 425 million and predicted to reach 629 million by 2045 worldwide [1]. Hence, The identification of novel molecules regulating glucose metabolism might provide new therapeutic targets for the treatment of T2D.

Betatrophin, also known as TD26 [2], refeeding induced fat and liver (RIFL) [3], lipoprotein lipase inhibition (lipasin) [4], and atypical angiopoietin-like protein 8 (ANGPTL8) [5], is primarily secreted by the human liver and white adipose tissue under the conditions of insulin resistance [6]; here we refer to it as ANGPTL8/betatrophin. ANGPTL8/betatrophin has been implicated in both glucose and triglyceride metabolism [7, 8]. It has been reported to improve glucose tolerance in mice by promoting β cell proliferation in response to insulin resistance [6, 9], which has raised hopes for seeking a novel therapeutic target to treat diabetes. However, subsequent studies have led to conflicting claims about its physiological roles in promoting β cell proliferation [10, 11]. These findings suggested that ANGPTL8/betatrophin was involved in triglyceride metabolism rather than glucose homeostasis [12]. Systematic review and meta-analysis indicated a preference for association between betatrophin and T2DM [13–15]. Hence, the role of ANGPTL8/betatrophin in β cell proliferation and glucose metabolism seems controversial. We do not yet know the mechanism of betatrophin action, which points to the importance of producing recombinant betatrophin and testing it directly for the effects on β cell mass [6]. In this study, recombinant ANGPTL8/betatrophin was successfully expressed in *Escherichia coli*, and the biological activity of the purified protein was tested in vitro.

## Materials and Methods

### Vectors, Strains, Cell Lines and Reagents

*Escherichia coli* cloning vector pEASY-T1, expression vector pEASY-E2, competent cells Trans1-T1 and Transetta (DE3) were purchased from TransGen Biotech (Beijing, China). The whole gene of the mature form of ANGPTL8/betatrophin was synthesized by Inovogen Tech. Co. (Beijing, China) into pUCE plasmids. The MIN6 cell line was obtained from iCell Bioscience Inc. (Shanghai, China). The HepG2 cell line was donated by Dr. Hu Xiaoyuan (Biotechnology Research Institute, Chinese Academy of Agricultural Sciences). The triglyceride enzyme assay kit was purchased from Applygen Technologies Inc. (Beijing, China). All other chemicals and reagents, unless otherwise stated, were purchased from Beijing Solarbio Science and Technology Co., Ltd. (Beijing, China).

### Construction of the Expression Plasmid, pEASY-E2-Betatrophin

Full-length mature form DNA of human ANGPTL8/betatrophin (555 bp) was obtained through PCR with pUCE-betatrophin plasmid as templates. The gene-specific primer pair was list as follows: Betatrophin-F: 5′-ATGGCTCCAATGGGTGGTCCAGAAT-3′, Betatrophin-R: 5′-CCTAGGTTAATGGTGATGGTGATGG-3′. It was synthesized by Sangon Biotech Co., Ltd. (Shanghai, China). The PCR product was separated by 1% agarose gel electrophoresis and purified by a gel extraction kit. Subsequently, it was cloned into the pEASY-E2 expression vector by TA cloning which was then transformed into *E. coli* trans1-T1 clone strains. The constructed plasmid pEASY-E2-betatrophin was abstracted and used as PCR templates. The PCR products were confirmed by gel electrophoresis as well as DNA sequencing. Then the plasmid was again transformed into *E. coli* Transetta (DE3) strain. Transetta (DE3)/pEASY-E2-betatrophin was used as engineering strain for the following work.

### Expression and Confirmation of Recombinant ANGPTL8/Betatrophin by SDS-PAGE and Western Blotting

The Transetta (DE3)/pEASY-E2-betatrophin strain was cultured in 10 mL Luria–Bertani (LB) medium with 50 mg/L ampicillin at 37 °C to an OD_600_ = 0.6–0.8. One mmol/L isopropy-β-d-thiogalactoside (IPTG) was added into the culture and incubated for approximately 6–8 h. Cells were harvested and ultrasonicated in phosphate buffer saline (PBS) (137 mmol/L NaCl, 2.7 mmol/L KCl, 10 mmol/L Na_2_HPO_4_, 2 mmol/L KH_2_PO_4_, pH 7.4). After centrifugation at 4 °C, 12,000 rpm for 10 min, both supernatant and insoluble fractions were collected and separated by sodium dodecyl sulfate–polyacrylamide gel electrophoresis (SDS-PAGE) with coomassie blue R-250 staining. The proteins separated on the SDS-PAGE gels were transferred onto a polyvinylidene fluoride (PVDF) membrane (Sigma-Aldrich, United States). Subsequently, western blotting was performed using goat anti-human betatrophin polyclonal antibody (A00528-11-100, Aviscera Bioscience, Inc., Santa Clara, USA) in 1:5000 dilution and rabbit anti-goat immunoglobulin G-alkaline phosphatase (IgG-AP, ab6742, Abcam Ltd., Cambridge, United Kingdom) in 1:3000 dilution visualized with 5-bromo-4-chloro-3-indolyl phosphatase/nitroblue tetrazolium chloride (BCIP/NBT) solution.

### Optimizing the Expression Conditions of ANGPTL8/Betatrophin

The optimal conditions for ANGPTL8/betatrophin expression, including IPTG concentration, induction time and temperature, were determined prior to large-scale production. The recombinant *E. coli* strain Transetta (DE3) carrying the pEASY-E2-betatrophin construct was induced with 0, 0.1, 0.3, 0.5, 0.8, 1.0, or 1.2 mmol/L IPTG at 200 rpm, 37 °C, for 8 h. The optimum IPTG concentration was determined using SDS-PAGE analysis visualized by coomassie blue R-250 staining. The Transetta (DE3)/pEASY-E2-betatrophin strain was then induced using the determined optimum concentration of IPTG at 200 rpm, 37 °C, for 0, 1, 2, 4, 6, 8, 10, or 12 h. Finally, the Transetta (DE3)/pEASY-E2-betatrophin strain was induced at 200 rpm using the optimum IPTG for an optimal time at 16, 30, or 37 °C.

### Large Scale Production and Purification of Recombinant ANGPTL8/Betatrophin

The engineered bacterial strain Transetta (DE3)/pEASY-E2-betatrophin was cultured in 500 mL LB medium with 50 mg/L ampicillin at 37 °C. When the OD_600_ was reached 0.6–0.8, 0.3 mmol/L IPTG was added into the culture and incubated for 8 h. Cells were harvested and disrupted by ultrasonication after resuspension in PBS buffer. Following centrifugation at 15,000 rpm for 30 min at 4 °C, inclusion bodies were collected and dissolved in 8 mol/L urea solution. Purification was performed using nickel–nitrilotriacetic acid (Ni–NTA) bind resin in a His-tagged affinity chromatography column (Qiagen, Hilden, Germany). Fusion proteins were then subjected to gradient dialysis for renaturation. The dry powder of the purified betatrophin-His fusion protein obtained from lyophilization was kept at 4 °C for further use.

### HPLC Analysis

High-performance liquid chromatography (HPLC) assay was performed by use of LC-2010A/C chromatographic instrument (Shimadzu, Japan) with a C18 column (250 × 4.6 mm, I.D.S-5 μm, 12 nm, YMC-Pack ODS-A, Germany). The mobile phase was acetonitrile/water (90:10, v/v) with 0.1% trifluoroacetic acid (TFA). The gradient assay was performed at ambient temperature at a flow rate of 1.0 mL/min and lasted for 20 min. A commercial betatrophin standard (00528-01-100, 100 μg, Aviscera Bioscience, USA) and a blank of PBS (pH 7.4) were used as controls. All samples were detected by measurements at 280 nm.

### Cell Proliferation Assay

To determine the promoting effect of *E. coli*-derived betatrophin on cell proliferation, a mouse pancreatic β cell line, MIN6, was used to evaluate the effect of recombinant betatrophin (r-betatrophin) on cell proliferation. Cells were cultured in Roswell Park Memorial Institute (RPMI) 1640 medium (Gibco, USA) with 10% (v/v) fetal bovine serum (FBS), 2 mmol/L l-glutamine, 50 μmol/L 2-mercaptoethanol, 100 U/mL penicillin and 0.1 mg/mL streptomycin at a high (95%) relative humidity (RH) and 5% CO_2_ at 37 °C. At a density of 3 × 10^4^ cells per well, MIN6 cells were seeded into a flat-bottomed 96-well microtiter plate. Twenty-four hours later, cells were washed twice with Dulbecco’s phosphate buffered saline (DPBS, Corning, USA). Subsequently, purified r-betatrophin at a concentration of 1.5, 5, 10, 15, or 25 μmol/L was added into the culture medium (n = 6). After 48-h incubation, 10 μL Cell Counting Kit-8 (CCK-8, Dojindo, Japan) was added to each well according to the manufacture’s instructions. CCK-8 kit utilizes the water-soluble tetrazolium salt-WST^®^-8 (2-(2-methoxy-4-nitrophenyl)-3-(4-nitrophenyl)-5-(2, 4-disulfobenzene)-2H-tetrazole mono-sodium salt) developed by Dojindo. The orange yellow formazan dye produced by the oxidation and reduction of WST^®^-8 by intracellular dehydrogenase can be dissolved in culture medium, and the amount of formazan produced is proportional to the number of living cells. So cultures were incubated for an additional 1–4 h. Finally, cell viability of all samples was analyzed in a microplate reader at 450 nm wavelength. The experiment was repeated twice for reproducibility.

### Measurement the Concentration of Triglyceride

HepG2 cells were treated with 200 nmol/L r-betatrophin for 48 h (n = 3). The cultures were then collected for determination of the triglyceride (TG) concentrations using a Triglyceride Quantification Assay Kit (E1003, Applygen Technologies Inc., China) according to the manufacturer’s instructions. The principle of the kit is as follows: (1) triglyceride was decomposed into glycerol by lipase; (2) glycerol was phosphorylated to glycerol-3-phosphate by glycerol kinase; (3) glycerol 3-phosphate was oxidized by glycerol phosphate oxidase to produce hydrogen peroxide and under the action of peroxidase, the chromogenic substrate was converted to benzoquinone. The color would be stabilized within 60 min after the reaction was equilibrated. The optical density value was proportional to the triglyceride concentration and the value of each tube was measured at 550 nm wavelength. Drew a standard curve and then calculated the triglyceride concentration. The experiment was repeated twice for reproducibility.

## Results

### Construction of Expression Plasmid pEASY-E2-Betatrophin

**Fig. 1:**
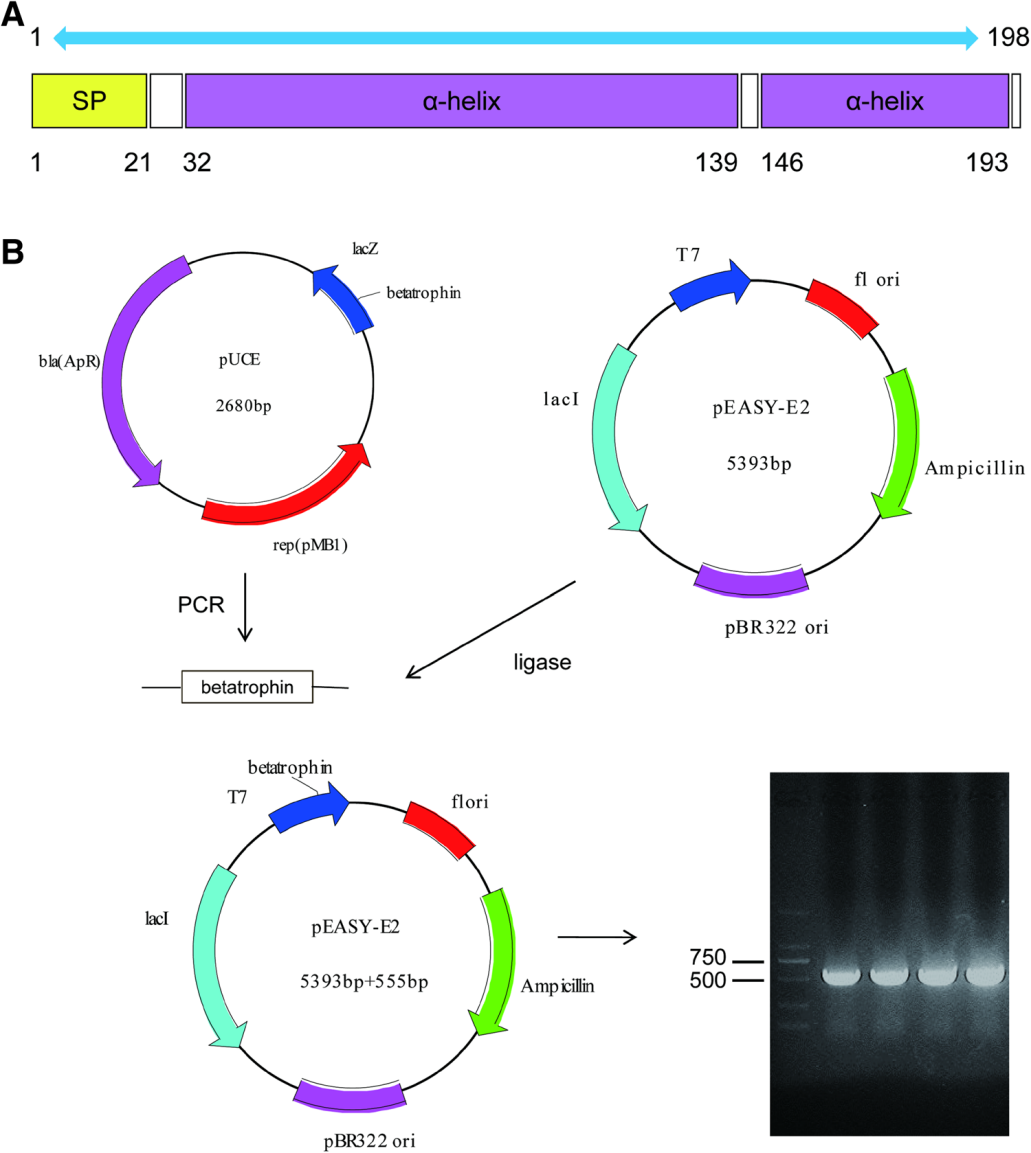
Construction and validation of the expression vector pEASY-E2-betatrophin. **a** Protein structure of ANGPTL8/betatrophin. *SP* signal peptide. **b** Construction and identification of the expression vector pEASY-E2-betatrophin. Lane 1: DNA Marker; Lanes 2–3: PCR products using Transetta (DE3)/pEASY-E2-betatrophin clones as PCR templates; Lane 4: positive control, using pUCE-betatrophin plasmid as the PCR template

Full-length DNA of mature form of human ANGPTL8/betatrophin (177 amino acids) was synthesized into vector pUCE. Using pUCE-betatrophin plasmid as the PCR template, 555 bp gene of fusion protein betatrophin-6 × His-tag was amplified with gene-specific primers. PCR products were then inserted into the purchased cloning vector pEASY-T1 by TA cloning and transformed into Trans1-T1. After PCR determination and sequence analysis, the gene of the fusion protein was amplified again using pEASY-T1-betatrophin plasmid as the PCR template. The products were purified and again inserted into the expression vector pEASY-E2 by TA cloning and transformed into Transetta (DE3) strain. The establishment process and the result of PCR determination using Transetta (DE3)/pEASY-E2-betatrophin clones as templates are shown in Fig. 1.

### Analyzing the Expression of Recombinant Betatrophin

**Fig. 2:**
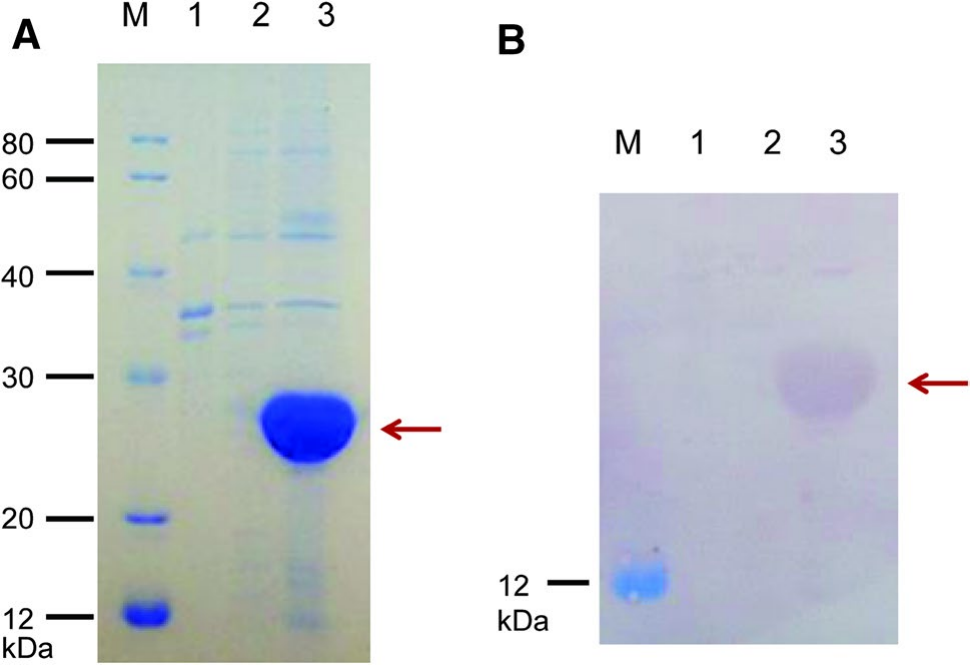
SDS-PAGE and western blot analysis of betatrophin. **a** Representative result of SDS-PAGE. **b** Representative result of western blot. M: protein marker; Lane 1: Transetta (DE3)/pEASY-E2-betatrophin strain without IPTG induction; Lanes 2 and 3: the supernatant and precipitate of Transetta (DE3)/pEASY-E2-betatrophin strain obtained from cell lysis and centrifugation following IPTG induction. Betatrophin is indicated by a red arrow (Color figure online)

The *E. coli* Transetta (DE3)/betatrophin strain was induced using IPTG for expression. Samples of supernatant and precipitate were obtained by centrifugation after collection and ultrasonication. The results of SDS-PAGE and western blotting analysis showed that r-betatrophin was rarely detected in the supernatant and highly expressed in the insoluble form of inclusion bodies (Fig. 2a, b). It indicated that the molecular weight (MW) of r-betatrophin was less than 25 kDa, which is consistent with the theoretically estimated MW of 22 kDa. It also indicated that the fusion protein was not degraded.

### Optimization of Recombinant Betatrophin Expression and Purification

**Fig. 3:**
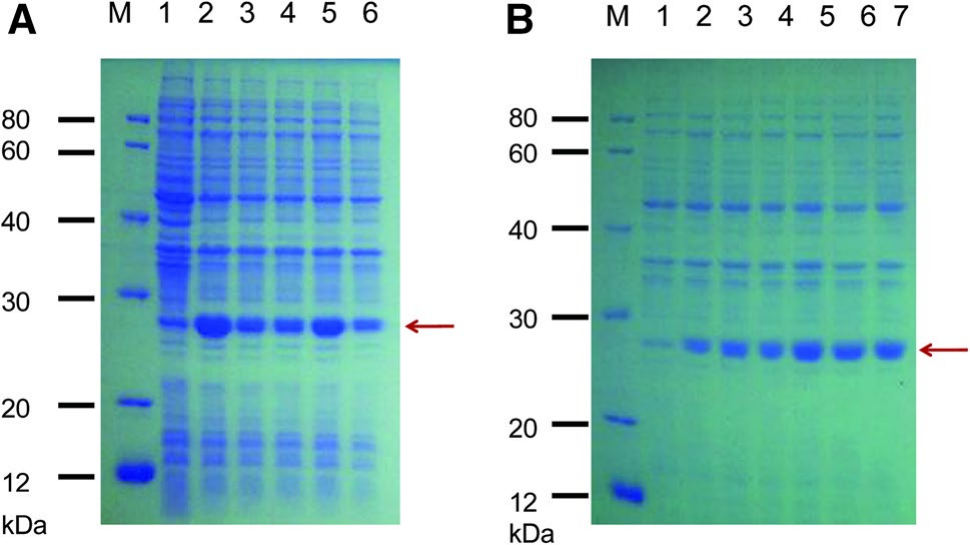
Optimizing prokaryotic expression conditions of betatrophin. **a** SDS-PAGE analysis of optimum IPTG concentration. M: Protein marker; Lanes 1–6: Transetta (DE3)/pEASY-E2-betatrophin strain induced by 0.1, 0.3, 0.5, 0.8, 1.0, and 1.2 mmol/L IPTG, respectively, at 30 °C, 200 rpm for 8 h. **b** SDS-PAGE analysis of optimum induction time. M: Protein marker; Lanes 1–7: Transetta (DE3)/pEASY-E2-betatrophin strain induced for 1, 2, 4, 6, 8, 10, and 12 h, respectively, with optimum IPTG concentration at 30 °C, 200 rpm. Recombinant betatrophin protein is indicated by a red arrow (Color figure online)

To maximize the output of r-betatrophin products, different concentrations of IPTG (0.1, 0.3, 0.5, 0.8, 1.0, or 1.2 mmol/L), culture time (1, 2, 4, 6, 8, 10, or 12 h) and temperatures (16, 30, or 37 °C) were employed. The SDS-PAGE analysis showed that the optimal concentration of IPTG was 0.3 mmol/L, and the optimal induction time was 8 h (Fig. 3a, b). However, even though the culture temperature was as low as 16 °C, little recombinant protein was present in the supernatant. Based on this, 30 °C was selected as the optimal culture temperature for the rapid growth and metabolic activity of *E. coli*.

**Fig. 4:**
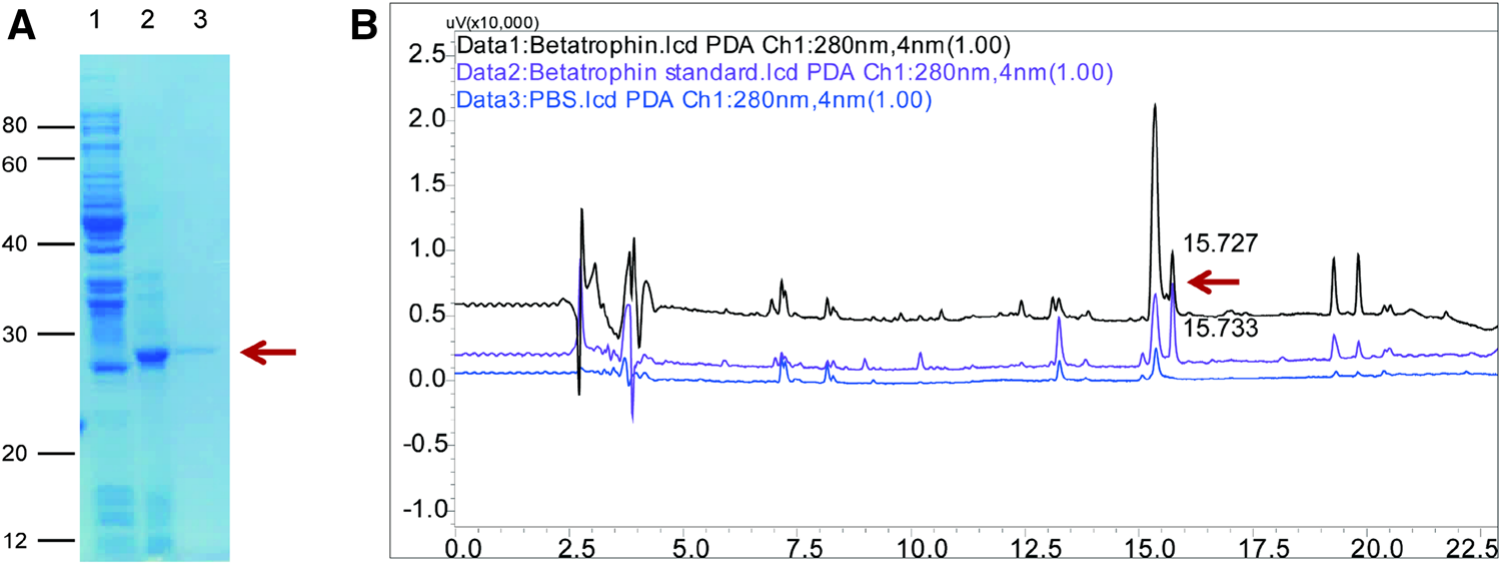
Purification analysis of r-betatrophin. **a** SDS-PAGE analysis of r-betatrophin with coomassie brilliant blue staining. Lane 1: Transetta (DE3)/pEASY-E2-betatrophin strain without IPTG induction; Lane 2: inclusion bodies form of r-betatrophin; Lane 3: dry powder of purified r-betatrophin reconstructed in PBS (pH 7.4). **b** HPLC analysis of r-betatrophin. Betatrophin is indicated by a red arrow (Color figure online)

Transetta (DE3)/pEASY-E2-betatrophin was induced under optimal expression conditions in order to obtain abundant r-betatrophin. After centrifugation following PBS (pH 8.0) resuspension, the inclusion bodies were solubilized using 8 mol/L urea in combination with ultrasonication when necessary. Subsequently, the soluble expression products were purified by Ni–NTA affinity chromatography. The fusion protein was then renatured by gradient dialysis to gradually remove urea via a graded series of PBS buffer (pH 7.4). After lyophilization, dry powder form of purified betatrophin was obtained. SDS-PAGE analysis demonstrated that r-betatrophin of high quality was successfully obtained (Fig. 4a). HPLC analysis indicated that the purity of r-betatrophin was approximately 100% (Fig. 4b). The retention time of the peak was 15.727 min, consistent with 15.733 min of a commercial standard, as shown in Fig. 4b. The yield of purified recombinant betatrophin was approximately 20 mg per liter of culture medium.

### Bioactivity Assay of Recombinant Betatrophin

**Fig. 5:**
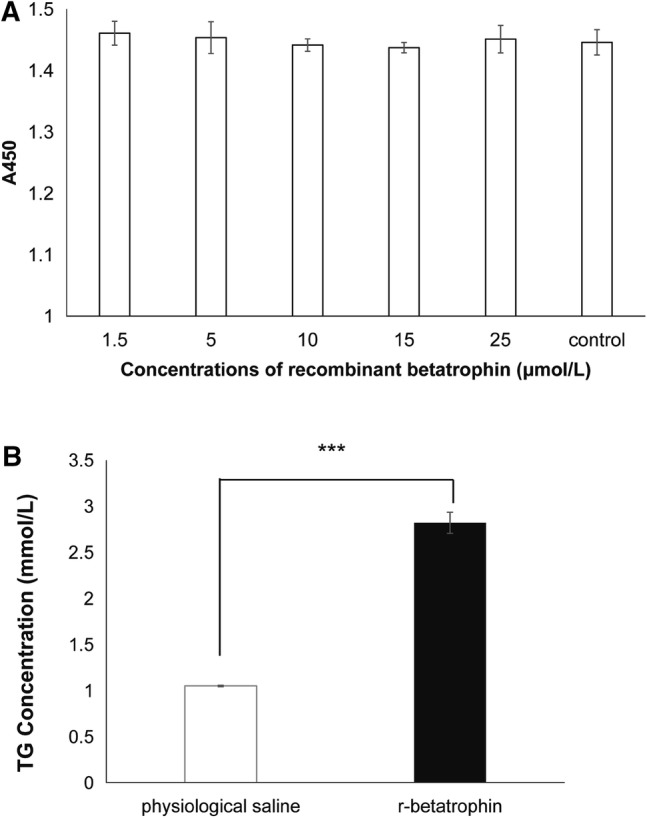
Biological activity of r-betatrophin. Effect of recombinant betatrophin on the proliferation of MIN6 cells (n = 6) (**a**) and TG matabolism of HepG2 cells (n = 3) (**b**). All experiments were repeated three times and “***” indicates *P* < 0.001

MIN6 cell line was used to detect the proliferation effect of betatrophin. In vitro, r-betatrophin was directly added to the cell culture at different concentrations, with DPBS-treated cells as the negative control. After incubation for 48 h, 10 μL CCK-8 solution were added into each well. One to four hours later, cell density was measured at 450 nm wavelength. The results demonstrated that compared with DPBS-treated controls, there was no significant difference in cell density for r-betatrophin-treated cells, indicating that betatrophin likely has no proliferation effect on β cells (Fig. 5a).

HepG2 cell line was used to detect the effect of betatrophin on TG metabolism. HepG2 cells were treated with 200 nmol/L r-betatrophin for 48 h. The cell cultures were then collected to measure TG levels. Our results showed that the concentrations of TG in the culture of r-betatrophin-treated cells were significantly higher than that of DPBS-treated cells (*P* = 0.002) (Fig. 5b).

## Discussion

ANGPTL8/Betatrophin is a newly characterized hormone which is primarily secreted by human liver and white adipose tissue. Till now, the function and mechanism of which is still unknown. It was initially reported to be involved in the control of β cell proliferation in mice response to insulin resistance [6]. Also, transplantation of adipose-derived mesenchymal stem cells expressing betatrophin induced human β cell proliferation [9]. However, subsequent studies revealed that ANGPTL8/betatrophin did not control pancreatic β cell expansion in *Angptl8* gene knockout/overexpression mice or mice transplanted with human β cells [10, 11, 16]. Moreover, overexpression of ANGPTL8 resulted in doubled plasma TG levels [10]. Multiple studies have indicated that betatrophin was involved in TG metabolism [4, 12, 17, 18]. Nevertheless, several groups independently found that the circulating betatrophin concentrations were correlated with insulin resistance or type 2 diabetes [14, 19–22]. Recently it has been reported to potentially be involved in hypertension [23], polycystic ovary syndrome [24–26], and urinary albumin excretion and renal function [27], which indicates it is a pleiotropic hormone.

The function of betatrophin is not clear and in some cases controversial. To understand the function of betatrophin in detail, it is necessary to make recombinant protein with high purity in adequate quantities.

In this study, we attempted to simplify the production procedure of recombinant betatrophin with high yield. The recombinant ANGPTL8/betatrophin was highly expressed in Transetta (DE3) cells, and the optimized expression conditions were induced with 0.3 mmol/L IPTG at 30 °C for 8 h. Ni–NTA His-bind Resin affinity chromatography was used for purification and HPLC for purity measurement. The entire production procedure is easy to implement with no need for specialized equipment. In vitro studies demonstrated that ANGPTL8/betatrophin did not promote MIN6 cell proliferation, but increased triglyceride levels in the culture of HepG2 cells, which supports the possibility that inhibition of ANGPTL8/Betatrophin represents a therapeutic strategy for hypertriglyceridemia [10]. Although the results of the biological assays were partially consistent with previous studies and showed that the recombinant betatrophin derived from bacteria was bioactive, the mechanism of it in the regulation of glucose metabolism is still unclear. As the dry lab revealed the regulatory role of ANGPTL8/betatrophin both in glucose and lipid metabolic pathways [28], further wet lab studies on its functional mechanism must be performed, and our procedure for obtaining active recombinant betatrophin in high quantities provides a material basis.
